# Application of the Differential Evolutionary Algorithm to the Estimation of Pipe Embedding Parameters

**DOI:** 10.3390/s22103942

**Published:** 2022-05-23

**Authors:** Ping Lu, Shuang Chen, Xiaozhen Sheng, Yan Gao

**Affiliations:** 1 State Key Laboratory of Traction Power, Southwest Jiaotong University, Chengdu 610031, China; 2 Rail Transit College, Chengdu Vocational& Technical College of Industry, Chengdu 610213, China; 3 School of Urban Railway Transportation, Shanghai University of Engineering Science, Shanghai 201620, China; shengxiaozhen@hotmail.com; 4 Key Laboratory of Noise and Vibration Research, Institute of Acoustics, Chinese Academy of Sciences, Beijing 100190, China; gaoyan@mail.ioa.ac.cn

**Keywords:** buried water pipe, pipe embedding parameters estimation, differential evolutionary algorithm, wavenumber estimation

## Abstract

The time-delay estimation (TDE) method is the primary method for predicting leakage locations in buried water distribution pipelines. The accuracy of TDE depends on the acoustic speed and attenuation of the leakage signal propagating along the pipeline. The analytical prediction model is the typical approach for obtaining the propagation speed and attenuation of leakage waves. However, the embedding parameters of the buried pipe in this model must be measured using soil tests, which are very difficult, costly, and time-consuming. These factors restrict the application of the TDE method in pinpointing pipeline leakage. A method for inverse identification of pipe embedding parameters using discrete wavenumbers obtained in field testing is presented in this paper, and the differential evolution algorithm is introduced as an optimization solution. A field experiment is conducted to validate the method, and the test wavenumbers are measured in a cast-iron pipeline. The estimated sensitive parameters in the analytical model using the method are soil elastic modulus, Poisson’s ratio, and pipe–soil contact coefficient, while the conventional soil test is used to measure the soil density due to the character of the optimization algorithm and the soil properties. The application effects show that the estimated parameters are close to those measured from a conventional soil test. The wave speed based on the estimated parameters was an excellent match for the on-site test in the engineering application. This work provides a less costly and more straightforward way to apply the TDE method for leak localization in buried pipelines.

## 1. Introduction

Water leakage, especially in buried pipelines, is a subject of increasing concern across the world because of the potential public health danger, economic constraints, environmental damage, and wastage of energy. Detecting and pinpointing leakage provides a key means to solving the issue. The leak detection methods in pipes can be classified into three categories [1]: methods based on signal processing, methods based on state estimation, and methods based on knowledge. Most methods based on signal processing focus on utilizing measurements collected from different sensors and applying different analytical techniques to detect and localize faults. The state estimation methods thus far focus on developing and using models based on fundamental principles to detect and localize leaks. Most of the methods based on knowledge were initially designed to detect leaks in systems with single flow. Among these methods, acoustic-based technology is more suitable for leak detection in water distribution pipelines.

Acoustic-based leak detection techniques have been in common use in water-distribution networks over the past 30 years [2,3]. They usually use the cross-correlation function between leak noise signals measured using hydrophones or accelerometers placed on both sides of the leak. The efficacy of a correlator depends upon knowledge of the speed at which the leak noise propagates along the pipe as well as how much it attenuates with distance. As is known, the fluid-borne wave in the pipe is the predominant energy-carrying mode in the pipe leakage detection field, and its propagating characteristics are profoundly influenced by the pipe and soil properties, especially in the pipe–soil strong coupling pipeline systems, e.g., buried plastic pipe systems. Muggleton [4] and Gao [5,6] observed that the medium outside the pipe acts on the pipe with additional mass and additional stiffness through the interface shear effect, affecting the acoustic speed and attenuation. Based on the analytical and finite element models, Brennan [7] further studied the effects of the soil properties surrounding the pipe on the propagation speed of the leakage acoustic wave, pointing out that the shear properties of the soil mainly affected the propagation speed of the acoustic wave, and the compression properties mainly affected the attenuation of the acoustic wave. The models were subsequently applied to two different types of soil—one sandy, the other clay—to validate the above study.

The theoretical model plays an important role in predicting leak wave speed and attenuation along the pipe. Although the pipe property parameters in this model can be determined relatively easily, estimation of the soil property parameters surrounding the pipe is more problematic. Representative soil samples—used for parameter testing—are difficult to obtain due to the large covering scale and the complex embedding conditions of the on-site pipeline. The subsequent time-consuming soil tests of the soil samples make them costly for practical applications. Meanwhile, the presumption of the theoretical model generally reduces agreement between predicted and actual wave propagating speed. Accordingly, in leakage-location engineering practice, a more accurate estimation method for soil properties is needed, along with a more convenient and precise method of determining propagating speed and attenuation of the leak wave.

Currently, in many industrial applications, the inversion identification of model parameters using field test data is an emerging approach to obtaining the soil parameters in a model [8,9]. Jesenik et al. [10] tested different soil models on measured data and used different metaheuristics to determine soil parameters. These inversion methods of soil parameters are worthy of reference, but the required test methods are not suitable for buried pipe conditions. Scussel et al. [11] introduced the idea of inversion into the buried liquid-filled pipe and used the cost-function algorithm to estimate the bulk modulus and shear modulus of the soil. However, the two above estimated parameters are not independent, which easily leads to multiple solutions. Meanwhile, it is easy to be trapped in local optima when solved by the cost-function algorithm for optimization problems with unclear gradient information.

An embedding parameters identification method for water-filled pipes based on the differential evolutionary algorithm is put forward using field test data. With this method, the pipe embedding parameters can be quickly inverted based on a few discrete leakage test data. The continuous speed in the full frequency band of interest can be obtained easily through the amended analytical model.

## 2. Method for Wavenumber Prediction

The leakage acoustic wave propagation has multiple modes and dispersion characteristics for the liquid-filled pipe system with a high coupling of pipe/soil. To predict the wave speed more accurately, Fuller [12] and Pinnington [13] proposed a wave-speed prediction analytical model for a liquid-filled pipe in a vacuum, which considered the dispersion characteristics for the modes of sound propagation along with the fluid and pipe. Muggleton [4] and Gao [5] proposed a theoretical model of wavenumber prediction considering the effect of external pipe medium on sound-wave propagation under the assumed extreme condition of lubricated contact and compact contact between pipe and soil. They then proposed the method for calculating the propagation speed of buried liquid-filled pipe, meanwhile observing that the fluid-dominated axisymmetric wave is the primary mode in leak detection.

Lu et al. [14] introduced the pipe–soil contact state variable into the fluid-dominated axisymmetric wave equations for the first time, which broke through the assumption of the extreme buried conditions, and the general wave equation of the axisymmetric wave of the buried liquid-filled pipe is obtained as follows:

In the above equations, the parameters of the pipe and fluid are easily available. The factors needing identification in the system are usually the soil parameters, which include Young’s modulus, Poisson’s ratio, density, and contact coefficient between pipe and soil. The first three parameters are generally obtained through conventional soil tests. The main on-site test methods for soil density are the ring knife method and the irrigation method, which are both mature technology [15]. The Poisson’s ratio is obtained by measuring the volume change, and is difficult to test accurately. The determination of the elastic modulus requires a high production level of instrument and equipment [16], which are not easy to operate in practice. Meanwhile, the determination method for the deformation modulus and shear modulus is easier to realize. The actual elastic modulus of the soil is often converted from the bulk modulus and shear modulus, and the conventional test method for both is the three-axis test [17]. At present, there is no suitable test technology for the contact coefficient of pipe and soil interface, which can only be gained by experience.

(1) $$k_{1}^{2}=k_{f}^{2}(1+\frac{\beta }{1-\Omega^{2}+\alpha }),$$

where:

(2) $$\left\{ \begin{array}{l} \alpha =-SL_{22}-\frac{\left[v_{p}+iSL_{12}/k_{1}a][v_{p}-i\xi SL_{21}/k_{1}a\right]}{1-\xi SL_{11}/k_{1}a}\\ \beta =2\frac{a}{h}\left(\frac{1-v_{p}^{2}}{E_{p}}\right)B_{f} \end{array}\right.,$$

where α stands for the surrounding medium loading and pipe parameters, which can be used to evaluate the influence of soil load on the pipe wall displacement, β refers to fluid and pipe parameters that can be used to evaluate the influence of fluid load on the pipe wall displacement, Ω is the non-dimensional frequency, $\Omega =\omega a/c_{L}=k_{L}a$, $k_{f}=\omega /c_{f}$ is the fluid wavenumber, $c_{f}=\sqrt{B_{f}/\rho_{f}}$ is the free-field fluid wave speed, $c_{L}$ is the shell compressional wave speed, $k_{L}$ is the shell compressional wavenumber, and $k_{fs}^{r}$ is the internal fluid radial wavenumber, which can be expressed as ${\left(k_{fs}^{r}\right)}^{2}=k_{f}{}^{2}-k_{s}{}^{2}$.

(3) $$\left\{ \begin{array}{l} SL_{11}=-\mu_{m}\frac{\left(1-v_{p}^{2}\right)}{E_{p}}\frac{a}{h}\frac{k_{ds}^{r}ak_{rs}^{r}a^{2}}{k_{rs}^{r}ak_{ds}^{r}a\left[H_{0}\left(k_{rs}^{r}a\right)/H_{0}^{\prime }\left(k_{rs}^{r}a\right)\right]+k_{s}^{2}a^{2}\left[H_{0}\left(k_{ds}^{r}a\right)/H_{0}^{\prime }\left(k_{ds}^{r}a\right)\right]}\\ SL_{12}=i\mu_{m}\frac{\left(1-v_{p}^{2}\right)}{E_{p}}\frac{a}{h}k_{s}a\left\{2-\frac{k_{r}^{2}a^{2}H_{0}\left(k_{ds}^{r}a\right)/H_{0}^{\prime }\left(k_{ds}^{r}a\right)}{k_{rs}^{r}ak_{ds}^{r}a\left[H_{0}\left(k_{rs}^{r}a\right)/H_{0}^{\prime }\left(k_{rs}^{r}a\right)\right]+k_{s}^{2}a^{2}\left[H_{0}\left(k_{ds}^{r}a\right)/H_{0}^{\prime }\left(k_{ds}^{r}a\right)\right]}\right\}\\ SL_{21}=SL_{12}\\ SL_{22}=-\mu_{m}\frac{\left(1-v_{p}^{2}\right)}{E_{p}}\frac{a}{h}\left\{2+\frac{k_{rs}^{r}ak_{rs}^{2}a^{2}\left[H_{0}\left(k_{rs}^{r}a\right)/H_{0}^{\prime }\left(k_{rs}^{r}a\right)\right]\left[H_{0}\left(k_{ds}^{r}a\right)/H_{0}^{\prime }\left(k_{ds}^{r}a\right)\right]}{k_{rs}^{r}ak_{ds}^{r}a\left[H_{0}\left(k_{rs}^{r}a\right)/H_{0}^{\prime }\left(k_{rs}^{r}a\right)\right]+k_{s}^{2}a^{2}\left[H_{0}\left(k_{ds}^{r}a\right)/H_{0}^{\prime }\left(k_{ds}^{r}a\right)\right]}\right\} \end{array}\right.,$$

$k_{ds}^{r},\;k_{rs}^{r}$ are the compression and shear wavenumbers of soil in the radial direction respectively, which can be expressed by compressed wavenumber $k_{d}^{}$, shear wavenumber $k_{r}^{}$, and wavenumber in the axil direction $k_{s}^{}$ as follows:

(4) $$\left\{ \begin{array}{l} {\left(k_{ds}^{r}\right)}^{2}={k_{d}}^{2}-{k_{s}}^{2}\\ {\left(k_{rs}^{r}\right)}^{2}={k_{r}}^{2}-{k_{s}}^{2} \end{array}\right.,$$

(5) $$\left\{ \begin{array}{l} k_{d}{}^{2}=\rho_{m}\omega^{2}/(\lambda_{m}+2\mu_{m})\\ k_{r}{}^{2}=\rho_{m}\omega^{2}/\mu_{m} \end{array}\right.,$$

$\lambda_{m},\mu_{m}$ are Lame coefficients, and $\rho_{m}$ is the density of the medium.

(6) $$k_{L}=\omega^{2}\rho_{p}\left(1-v_{p}{}^{2}\right)/E_{p},$$

(7) $$\left\{ \begin{array}{l} \lambda_{m}=E_{m}v_{m}/(1+v_{m})(1-2v_{m})\\ \mu_{m}=E_{m}/2(1+v_{m}) \end{array}\right.,$$

ξ∈[0,1] represents the contact coefficients to the actual boundary conditions at the pipe–soil interface, among which ξ=1 represents compact contact, and ξ=0 represents lubrication contact. The other parameter details in Equations (1)–(7) are given in reference [14].

In the above equations, the parameters of the pipe and fluid are easily available. The factors needing identification in the system are usually the soil parameters which include Young’s modulus, Poisson’s ratio, density, and contact coefficient between pipe and soil. The first three parameters are generally obtained through conventional soil tests. The main on-site test methods for soil density are the ring knife method and the irrigation method, which are both mature technology [15]. The Poisson’s ratio is obtained by measuring the volume change, and is difficult to test accurately. The determination of the elastic modulus requires a high production level of instrument and equipment [16], which are not easy to operate in practice. Meanwhile, the determination method for the deformation modulus and shear modulus is easier to realize. The actual elastic modulus of the soil is often converted from the bulk modulus and shear modulus, and the conventional test method for both is the three-axis test [17]. At present, there is no suitable test technology for the contact coefficient of pipe and soil interface, which can only be gained by experience.

By Equation (2), β can be obtained directly, but α, related to the unknown wavenumber $k_{1}$, cannot be solved directly. As it is difficult to obtain the closed analytical solution of Equation (1), the numerical method is adopted by transforming the differential equation solving problems into optimization problems.

**Figure 1:**
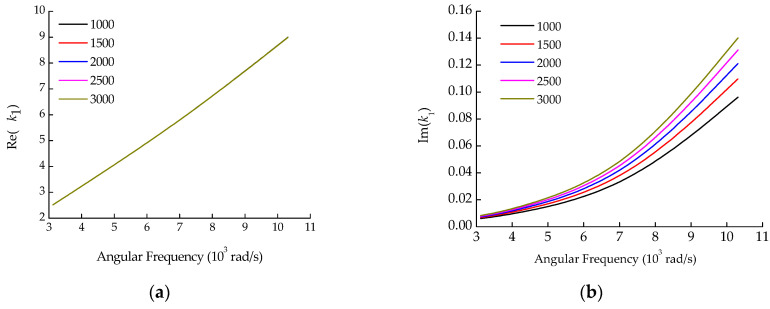
The influence of soil density on wavenumber. (**a**) Real part; (**b**) Imaginary part.

**Figure 2:**
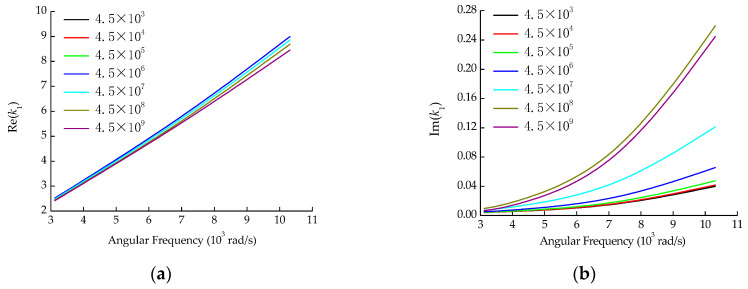
The influence of soil elastic modulus on wavenumber. (**a**) Real part; (**b**) Imaginary part.

**Figure 3:**
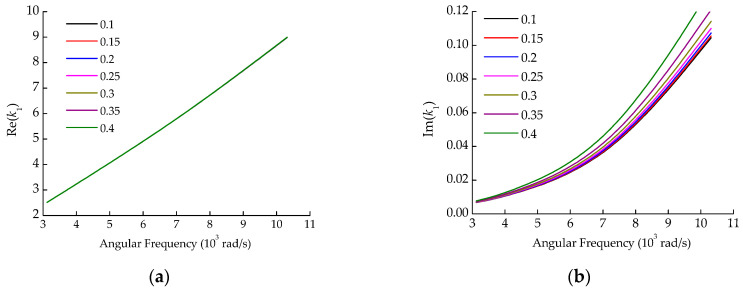
The influence of Poisson’s ratio on wavenumber. (**a**) Real part; (**b**) Imaginary part.

The above theoretical model can be solved using the Nelder–Mead method [18] to obtain the wavenumber information. To pick out effective design variables in the algorithm in Section 3, the sensitivity of the wavenumber was analyzed for each parameter, as shown in Figure 1, Figure 2 and Figure 3. The parameters of the pipe system are shown in Table 1.

It can be seen from Figure 1, Figure 2 and Figure 3 that the effects of soil density and Poisson’s ratio on the wavenumber are similar, both having a considerable influence on the imaginary part, but little influence on the real part. The elastic modulus affects both the real and imaginary parts of the wavenumber, especially when the elastic modulus increases above 4.5 × 10^7^ N/m^2^. From the optimization algorithm viewpoint, the design variables with similar influences on the objective function are likely to produce thematical multiple solution problems. Thus, soil density and Poisson’s ratio could not be used simultaneously as design variables for the optimization algorithm. Density is relatively stable and easy to measure, while Poisson’s ratio is the physical parameter that is not easy to measure accurately by the current test means. Therefore, the Poisson’s ratio was taken as the identification target parameter of the optimization algorithm, while the density was still determined by the conventional soil test method.

## 3. The Estimation Method of Pipe Embedding Parameters

According to the theory of wavenumber prediction, there is a mapping relationship between pipeline embedding parameters and wavenumbers. When the wavenumbers corresponding to some frequencies are obtained by field testing, theoretically, the pipe embedding parameters approaching the test wavenumber can be obtained through mathematical optimization.

Traditional mathematical optimization methods rely on the derivative or gradient matrix of each iteration step to determine the next step’s search direction and step length. However, the above theoretical wavenumber is not directly expressed by the wave equation but obtained by numerical methods, which leads to the difficulty in obtaining a gradient matrix, and even to singularity. Consequently, the methods dependent on gradient information are difficult to use directly for pipeline embedding parameter identification.

Due to the particularity of the engineering problems, optimization algorithms that do not require a gradient matrix are widely adopted. The EM algorithm is a commonly used tool for estimating the parameters for a mixture model but is more dependent on the initial values [19]. Bio-inspired optimization that does not need to iterate with a gradient matrix and does not depend on the initial values is a growing research topic to solve large-scale complex optimization problems. Jesenik, et al. [20] employed bio-inspired methods to determine a DC motor and drive parameters. Due to its huge computational amount, complex structures and many parameters are needed to control the bio-inspired algorithm in the application. LaTorre et al. [21] proposed methodological guidelines to prepare a successful proposal through many surveys. Liu et al. [22] introduced a parameter control approach utilized as feedback to control evolution processes adaptively.

Genetic algorithm (GA), particle swarm optimization (PSO), and differential evolution algorithm (DE) are very excellent bio-inspired optimization processes; however, each would need to be adapted according to the actual engineering problem. DE was chosen mainly because the algorithm does not have patent protection, which means it is possible to popularize the soil parameter estimation method. DE was put forward by Storn and Price [23] in 1995 and has gained wide applications [24,25,26].

DE is a parallel direct search method that utilizes NP dimensional parameter vectors as population X for each generation. In searching the optimal solution, two-parent vectors were selected and subtracted to obtain the differential vector. DE generates new parameter vectors by adding the weighted difference between two population vectors to a third vector, called the mutation operation. Then, the mutated vectors are mixed with the parameters of another predetermined vector to yield the trial vector. If the trial vector yields a lower cost function value than the target vector, the trial vector replaces the target vector in the following generation, which is called the selection operation. Through the several-generation evolution of mutation, crossover, and selection operation, the optimal individuals are retained, inferior individuals are eliminated, and the population is guided to approach the optimal value gradually.

According to the analysis in Section 2, the parameters to be identified are the Elastic modulus E, Poisson’s ratio v of the medium surrounding the pipe, and the contact coefficient of pipe and soil *ξ*, which constitute the vectors of the optimized design variables x:

(8) x=(E,v,ξ).

The parameters based on their physical meaning are set as:

(9) $$\{\begin{array}{l} x_{\min}=[1\times 10^{3},0,0]\\ x_{\max}=[1\times 10^{10},0.5,1] \end{array}.$$

NP sets of initial values are randomly generated within the constraints of the vector, which form a population X:

(10) $$X=[x_{1},x_{2},\cdots ,x_{NP}],$$

The approximation of the wavenumber corresponding to the target value of each group of the individual vector was calculated using the function *f*:

(11) $$f(E,v,\xi )=\sqrt{{\left(w_{\mathrm{re}}f_{\mathrm{re}}\right)}^{2}+{\left(w_{\mathrm{im}}f_{\mathrm{im}}\right)}^{2}}\to \min.$$

Since the wavenumber is complex, it is commonly difficult to simultaneously achieve the same degree of approximation in real and imaginary parts. Therefore, the weight coefficient $w_{re}$, $w_{\mathrm{im}}$ is introduced to consider the relative importance of the real part $f_{re}$ and the imaginary part $f_{\mathrm{im}}$:

(12) $$\left\{ \begin{array}{l} f_{\mathrm{re}}(E,v,\xi )=\sum_{i=1}^{M}\frac{\left|k_{\mathrm{re}}{}^{tar}(\omega_{i})-k_{\mathrm{re}}{}^{calc}(\omega_{i})\right|}{Mk_{\mathrm{re}}{}^{tar}(\omega_{i})}\\ f_{\mathrm{im}}(E,v,\xi )=\sum_{i=1}^{M}\frac{\left|k_{\mathrm{im}}{}^{tar}(\omega_{i})-k_{\mathrm{im}}{}^{calc}(\omega_{i})\right|}{Mk_{\mathrm{im}}{}^{tar}(\omega_{i})} \end{array}\right.$$

where *M* is the number of frequencies requiring calculation, *k^tar^* is the target wavenumber, and *k^calc^* is the calculated wavenumber in the current step.

After several generation populations of mutation, crossover, and selected operation, the function *f* approximates to a minimum. When the smallest function value *f* in the population no longer decreases in the subsequent *t* cycles, the loop will exit, and the exit condition can be expressed as:

(13) $$\left|\min f_{g}-\min f_{g+t}\right|<\varepsilon ,$$

where *g* is the current number of cycles, termed the generation number, and ε is the tolerance error.

The final identified pipe embedding parameters are the vector corresponding to the individual minimizing the function *f* in the last population. The solution process is shown in Algorithm 1.
**Algorithm 1** Pseudo-Code for the DE Process1.  set control parameters *NP*, *CR*, ε.2.  randomly generate the initial population of vectors in 3-dimensional search space3.  *g* = 14.  **repeat**5.  **for each** individual *j* in the population **do**6.    // begin mutation operation7.    *F* = 2 − *g*/3008.    select three mutually exclusive random individuals $x_{r1}$, $x_{r2}$, $x_{r3}$
9.    where, r1≠r2≠r3
10.     generate a donor individual by Equation (14)11. (14)$$b_{j}=x_{r1}+F(x_{r2}-x_{r3})$$12.    // end mutation operation13.    **do**14.       **while** (g ≤ 300 **AND** Equation (13) is false)15.       // begin crossover operation16.       *m* = a random integer in the range of [1, 3]17.       randD = [1, 2, 3]18.       generate a trial individual $u_{j}$ employing crossover by Equation (15)19.
(15)$$\;u_{jq}=\{\begin{array}{l} b_{jq},\;\mathrm{if}\;\mathrm{rand}(m)\;\leq \;CR\;\mathrm{or}\;\mathrm{randD}(q)\;=\;m,\;q\in [1,\;2,\;3]\;\\ x_{jq},\;\mathrm{for}\;\mathrm{all}\;\mathrm{other}\;\mathrm{dimensions} \end{array}$$
20.       // end crossover operation21.       // begin selection operation22.       evaluate the candidate individual $u_{j}$ using Equation (11)23.       replace $x_{j}$ with $u_{j}$, if fitness of $u_{j}$ is better than fitness of $x_{j}$
24.        // end selection operation25.       **end while**26.    *g* = *g* + 127.  **end for**

In Algorithm 1, *j* is the individual sequent number, and *j* ∈ [1, *NP*]; $b_{j}$ is the mutated individual, $u_{j}$ is the crossovered individual, and $x_{j}$ is the individual in current population, respectively; *F* is the amplification factor of the differential variation; rand(m) indicates generating a random value from [0, 1] in each dimension; **q** is a dimensional sequent of the individual vector; and *CR* is the crossover constant that influences computational efficiency and accuracy—in this paper, *CR* = 0.5.

Choosing a reasonable amplification factor *F* is essential for the algorithm to weigh the global convergence difficulty and computational efficiency. Larger values of *F* can help the function jump from the local optimum to the global optimum but deteriorate the algorithm’s convergence. Smaller values of *F* are in favor of the convergence; however, against the computational efficiency and prone to a local optimum. The elastic modulus as the design variable varies widely, and its effect on the objective function is non-monotonic. It is improper to use a traditionally constant value for *F*. A variable *F* is employed with the increasing generation number, termed *F* = 2 − *g*/300, and 300 is the maximum number of cycles in this paper. At the beginning of the algorithm’s operation, a particularly high value should be set to ensure the population’s approximation to the global optimum. In the following stages of the algorithm, the value of *F* becomes smaller to ensure convergence. *F* is a real and constant factor in a particular generation and varies with each generation. If a mutant individual exceeds the boundary values shown in Equation (9), the mutant individual will be replaced by the boundary values.

## 4. Application of the Method

**Figure 4:**
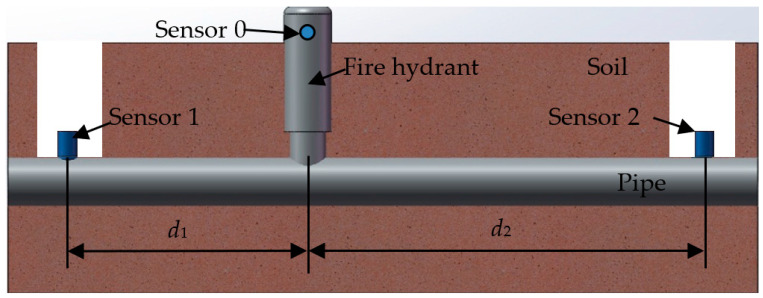
Schematic of the set-up for acoustic wave propagation estimation.

**Figure 5:**
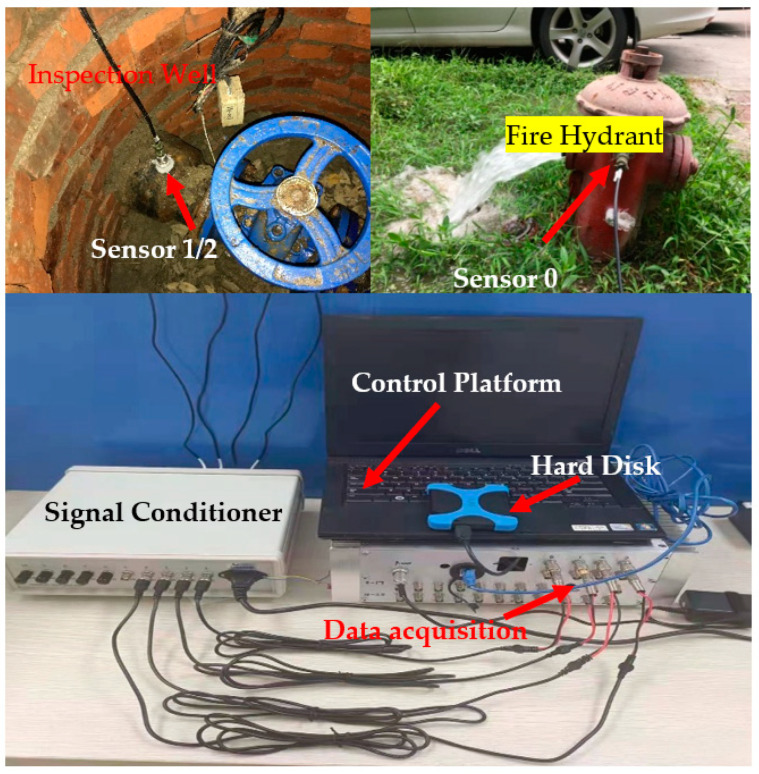
Test platform.

To validate the feasibility of the method, the cast-iron pipe embedding parameters were estimated using a set of wavenumbers obtained from the on-site test. Figure 4 shows a schematic diagram of the test of the acoustic wave propagation in a buried water pipe. The test platform is shown in Figure 5. Sensor 0 was installed on a hydrant to collect the vibration response in the normal direction caused by water discharge from the hydrant. Sensors 1 and 2 were installed on the pipe wall in the inspection wells on both sides of the hydrant to collect the leakage propagation signals.

The time delay between the two-way leak signals collected by Sensor 1 and Sensor 2 can be acquired using the cross-correlation analysis method. The corresponding wave speed and attenuation are easily available through the acquisition of the interval distance from the hydrant to either test point. Accordingly, the test can obtain wavenumbers at the characteristic frequencies, as shown in Table 2, which shows the target wavenumber *k^tar^* in Equation (12).

**Figure 6:**
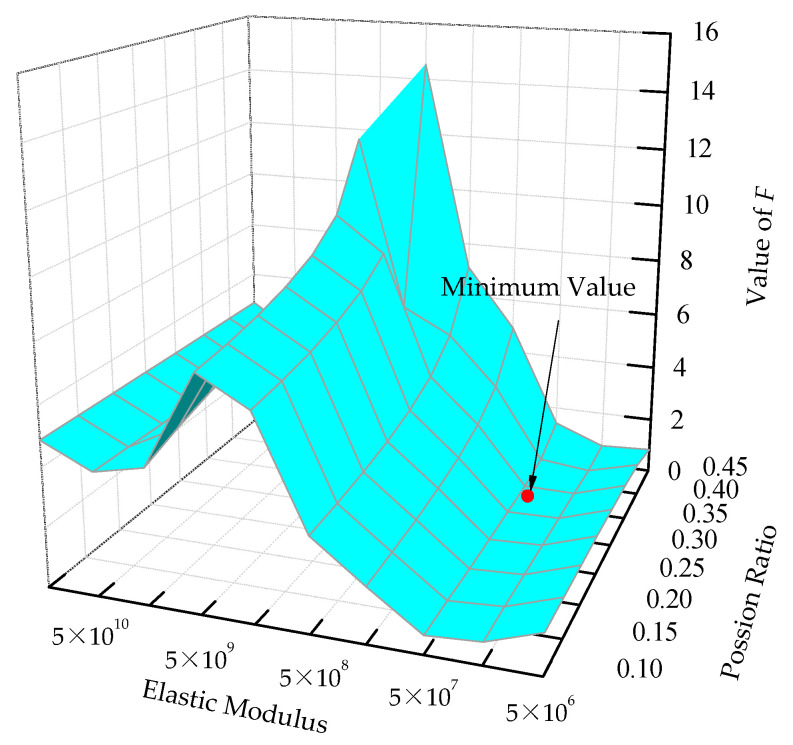
The variety of approximate functions with the identified soil parameters.

In DE calculation, each population vector serves once as the target vector, so that *NP* competitions occur in one generation. The larger the *NP*, the more computation in a single cycle; however, there are fewer total cycles with larger *NP*. Because the *NP* competitions within the same generation can be performed in parallel, *NP* should be set as a multiple of the number of computer threads. Moreover, it is also undesirable to significantly increase *NP* to reduce the cycles, as this would result in a longer overall calculation time. In this calculation, the number of computer threads is 10, setting *NP* as 50, and $w_{re}$ = 1, $w_{im}$ = 5 according to experience and convenience. Figure 6 shows the trend of the approximation function *f* with the elastic modulus and the Poisson’s ratio. The approximation function has a local minimum on each side of the elastic modulus of 1 × 10^9^ N/m^2^. The proposed algorithm randomly generates the initial population in the whole domain, which can effectively jump from the local optimum to the global optimum. The final estimate is the parameters corresponding to the minimum value of *f* in the figure.

Table 3 shows the respective values of the pipe embedding parameters obtained according to the conventional soil test and estimated method. As shown in the table, the values estimated by the inversion identification approach are very close to the soil test measurement. The deviation rate of the elastic modulus is about 6.44%, and the deviation rate of Poisson’s ratio is about 4.97%, meeting the accuracy requirements for soil engineering applications.

**Figure 7:**
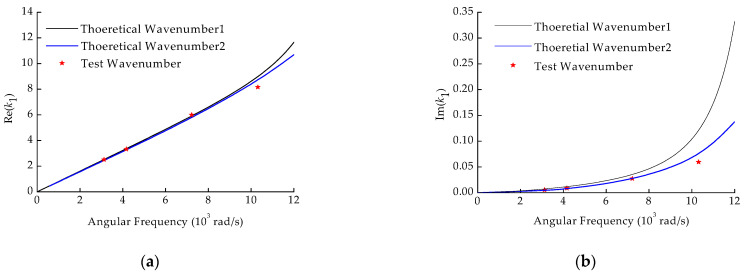
The comparison between the theoretical wavenumbers and the field test wavenumbers. (**a**) Real part; (**b**) Imaginary part.

The parameter estimation aims to provide a quick and accurate way to obtain the continuous wave speed in the full interest frequency domain through the wavenumber theoretical model. The soil test and estimated parameter values in Table 3 were each substituted into the theoretical formula in Section 2. Theoretical Wavenumbers 1 and 2 were obtained, as shown in Figure 7, and compared with the test wavenumber. The Wavenumber 1 curve represents the results calculated by soil test values, and the Wavenumber 2 curve represents those calculated by the estimated values. As is generally believed, the test wavenumber is the most accurate for water leakage pinpoint localization. There was a good consistency for the real part; however, there was a notable difference for the imaginary part between Wavenumber 1 and Wavenumber 2. Wavenumber 2 was closer to the test value for both the real and imaginary parts. 

The deviation rate of the two theoretical wavenumbers relative to the test wavenumber is shown in Table 4. The theoretical wavenumber deviation rate of the real part calculated using the estimated values of pipe embedding parameters was slightly higher than that calculated using the soil test values generally. The deviation rates were within 5.68%, meeting the engineering requirements. Meanwhile, regarding the deviation rate of the imaginary part, Theoretical Wavenumber 2 had a better performance. For the soil samples used in the soil test, it was difficult to fully represent embedding soil properties along the pipeline due to the inhomogeneity of the site soil. This may be one of the reasons for the large deviation of the imaginary part, as well as the real part at high-frequency in Theoretical Wavenumber 1. Since they were derived from on-site buried conditions, the estimated values of pipe embedding parameters adequately matched the on-site pipe buried situation in the engineering application.

## 5. Conclusions

An estimation method for pipe embedding parameters was put forward to improve the applicability of the wavenumber prediction theory model for leakage location. This method can quickly identify the sensitive pipeline embedding parameters according to the test wavenumber based on the differential evolutionary algorithm.

To ascertain the effective design variables in the algorithm from the pipe embedding parameters, an analysis of the sensitivity of the soil parameters to wavenumber was carried out in this work. Since the conventional soil test can easily measure the soil density, it was reasonable and feasible to choose the soil elastic modulus, Poisson’s ratio, and the pipe/soil contact coefficient as the design variables to be estimated.

The DE algorithm was briefly introduced in Section 3 of this paper, and the procedure of the DE algorithm was given considering the specific situation of the soil parameter identified. Control methods for population mutation and crossover in soil parameter estimation were given, and the feasibility of the method was validated based on the cast-iron pipeline field test. The outputs showed that the estimated parameters were very close to those obtained from the soil test, the deviation rate of the elastic modulus was about 6.44%, the Poisson’s ratio is about 4.97%, and the estimated pipe–soil contact coefficient is 0, which is consistent with traditional cast-iron pipes. Compared with the tested wavenumbers, the maximum deviation rate of the theoretical wavenumbers calculated with the estimated parameters was 5.68% for the real part and 19.12% for the imaginary part, which satisfies the leakage localization requirement in engineering applications.

According to the acoustic wave propagation theory of the buried liquid-filled pipe, the coupling effect of soil on the plastic pipe is enhanced, and wave propagating speed and attenuation are seriously affected by the surrounding medium. Future work will explore methods for estimating soil parameters for plastic pipes. Meanwhile, research on the application of this method to on-site pipe leak localization engineering will also be conducted.

## Figures and Tables

**Table 1:** Properties of the theoretical model for the cast iron pipeline.

Properties	Fluid	Pipe	Surrounding Medium
Density (kg/m^3^)	1000	7800	1999
Young’s modulus (N/m^2^)	-	1.22 × 10^11^	4.5 × 10^7^
Bulk modulus (N/m^2^)	2.25 × 10^9^	-	5.0 × 10^7^
Shear modulus (N/m^2^)	-	-	1.67 × 10^7^
Poisson’s ratio	-	0.25	0.35
Material loss factor	-	0.01	-

**Table 2:** The real and imaginary parts of the test wavenumber.

No.	Angular Frequency (rad/s)	Real Part of Wavenumber	Imaginary Part of Wavenumber
1	3121.9580	2.5080	0.00544
2	4167.0400	3.3276	0.00882
3	7221.7360	5.9966	0.02740
4	10,314.4800	8.1600	0.05960

**Table 3:** Comparison between the identified values and the test values for soil parameter.

Items	Elastic Modulus N/m^2^	Poisson Ratio	Contact Coefficient
Test Values	4.5 × 10^7^	0.35	-
Estimated Values	4.79 × 10^7^	0.3326	0

**Table 4:** The deviation rate relative to the test wavenumber.

No.	Angular Frequency (Hz)	Real Part of		Imaginary Part of	
		Wavenumber 1	Wavenumber 2	Wavenumber 1	Wavenumber 2
1	3121.9580	0.41%	2.60%	34.38%	19.12%
2	4167.0400	1.16%	1.85%	34.01%	18.37%
3	7221.7360	1.32%	4.36%	29.45%	13.47%
4	10,314.4800	10.17%	5.68%	103.29%	9.06%

## Data Availability

Not applicable.
